# *‘*People Like Us Would Have No Clue If the Information Is Online’: Exploring Understanding and Sources of Hepatitis B Information Among Vietnamese Australians

**DOI:** 10.1007/s40615-024-02055-4

**Published:** 2024-06-25

**Authors:** Loren Brener, Hoang Minh Khoi Vu, Robyn Horwitz, Elena Cama, Kacey Martin, Jake Rance, Sylvester Okeke, Joanne Bryant

**Affiliations:** Centre for Social Research in Health, UNSW Sydney, Sydney, Australia

**Keywords:** Hepatitis B, Knowledge, Health promotion message, Stigma, Cultural beliefs

## Abstract

Socio-cultural and behavioural factors are often not adequately considered in designing health promotion programs for culturally and linguistically diverse communities in Australia. Given that people of Vietnamese background are disproportionately impacted by hepatitis B, the aim of this research was to better understand these factors to inform hepatitis B health promotion messages for the Vietnamese community. Twenty participants (four living with hepatitis B) were interviewed by a Vietnamese-speaking researcher. The interview sessions explored beliefs about health, the body and liver; knowledge and attitudes about hepatitis B vaccines, testing, clinical management and stigma; and sources of health information and value given to information on social media. Participants had a range of understandings of health and hepatitis B which informed their responses to health education and intervention. Participants appeared to have limited knowledge and misconceptions about transmission, prevention, treatment, and management of hepatitis B. Stigma surrounding hepatitis B was apparent, with over half the participants reporting that they distanced themselves from people living with hepatitis B. Participants preferred online information resources for younger people and traditional media in the Vietnamese language for older people. By understanding what Vietnamese people know about hepatitis B and how they access health information, these findings can be used to inform health promotion campaigns using print, media, and radio to ensure wide reach. Knowledge of community specific information is key to reducing the burden of hepatitis B among culturally and linguistically diverse communities and ensuring they are able to access healthcare services for testing, monitoring, and care.

## Introduction

Hepatitis B is a blood-borne virus that can be transmitted vertically and horizontally [1]. Though preventable and treatable, globally, approximately 254 million people were living with chronic hepatitis B in 2022 with over 1 million new infections and 1 million annual deaths [2]. In Australia, about 200,385 people are living with hepatitis B, with 72.5% of this number being diagnosed [3] Australia has committed to targets set by the World Health Organisation (WHO) to eliminate hepatitis B as a public health threat by 2030 [4]. Some culturally and linguistically diverse (CALD) communities in Australia bear a greater burden of hepatitis B infection. Of the 200,385 people living with chronic hepatitis B in Australia in 2021, approximately 70% were born overseas, with the highest prevalence among people of CALD backgrounds from North-East and South-East Asia [3]. More than 6000 people, largely from CALD backgrounds, will be diagnosed with hepatitis B in Australia each year [5]. The Vietnamese-born population is the sixth largest CALD community in Australia over 257,997 Vietnamese-born people living in Australia [6]. Despite making up just 1% of Australia’s total population, Vietnam-born residents disproportionately account for an estimated 10.3% of chronic hepatitis B infections in Australia [3].

Health-related stigma––which connotes a social process involving devaluing people because of their perceived health conditions [7]––is a known factor impacting hepatitis B transmission and care. Stigma towards people living with hepatitis B stems from misinformation and a lack of knowledge of transmission routes, which leads to a fear of infection [8–13]. Stigma can also act as a barrier to healthcare access [10, 13–15]. People of CALD backgrounds may face additional issues—such as adapting to a new country, understanding the health system, racism, language barriers, history of trauma, family conflict, disengagement from services and mainstream society, low socio-economic status, unemployment, and insecure living arrangements—impacting willingness and ability to access health care, testing, treatment, and prevention care [16, 17]. Generally, healthcare access issues experienced by people of CALD backgrounds in high-income countries are largely related to social exclusion resulting from structural barriers, stigma, and discrimination [17–19]. Barriers to healthcare faced by people of CALD backgrounds, particularly accessibility and affordability, are often further compounded by fears that a positive blood-borne virus diagnosis may lead to visa cancellation or refusal of a visa in future [20, 21].

There is limited research on hepatitis B in the Australian Vietnamese community though some studies exist. Research in metropolitan Brisbane showed poor hepatitis B knowledge with 50% of survey participants being unaware that hepatitis B could be transmitted through unprotected sex and 68% believing it could be transmitted through sharing food and drinks [22]. Approximately one in ten in participants (11.6%) reported positive hepatitis B test results with only about half of this number (56%) currently on treatment [22]. Findings from another study that utilised semi-structured interviews with 22 Vietnamese people with chronic hepatitis B in Melbourne found that a main barrier to prevention and access to care is the broader social and cultural context in which hepatitis B occurs, which influences an individual’s understanding and response to hepatitis B [23]. Notably, the disconnect between biomedical interpretations of hepatitis B and the health beliefs of Vietnamese people living with hepatitis B underlies the misconceptions and lack of information surrounding hepatitis B transmission routes and treatment [23]. CALD people of Vietnamese background with hepatitis B also reported ‘living in constant fear of stigma and marginalisation’ which was unanimously attributed to ‘prevailing community misconceptions about hepatitis B-related transmission routes and disease outcomes’ [12]. These findings demonstrate a need for greater understanding of how to design, implement and evaluate responsive hepatitis B education, prevention, and care in Vietnamese CALD communities in Australia.

To achieve elimination targets and reduce the burden of hepatitis B disease in Australia by 2030, it is important to understand how hepatitis B is experienced and perceived amongst priority populations [4]. Hepatitis B treatment and prevention may be impacted by different social and cultural frameworks, particularly in CALD communities. There are a range of socio-cultural and behavioural factors that influence a person’s beliefs and experiences of hepatitis B that may contrast with the biomedical model of health, which fails to take account of the role of cultural understandings of health and illness [24]. People’s health beliefs and cultural values shape their health behaviour, including whether, where, and how they access healthcare services [24, 25].

Socio-cultural and behavioural factors are often not adequately considered in the design of health promotion programs [14]. These factors affecting hepatitis B risk behaviour and health service use are under-researched and often not well understood by health workers working with these culturally diverse populations. The aim of this research is thus to better understand these socio-cultural and behavioural factors and to contribute to the limited existing national and international literature on the attitudes and understanding of the Vietnamese community around hepatitis B. This study qualitatively explored understandings of health, attitudes towards hepatitis B, knowledge about vaccines, testing, and clinical management of hepatitis B, and experiences and impacts of stigma related to hepatitis B. The research also included a particular focus on establishing where members of the Vietnamese community seek health information, including access to different media sources, social media use, as well as which sources of health information were perceived to be valuable and trustworthy. These insights will help inform the design appropriate public health messages around hepatitis B that will be accessible, valued, and trusted by the Vietnamese community.

## Methods

### Data Collection

Between June and August 2022, the research team conducted a series of online consultation meetings with community organisations and key stakeholders as part of formative work to scope the research proposal, methods, and potential impact of the project. Promotional materials advertising the study in the Vietnamese language were posted to Facebook groups targeted at the Vietnamese community in New South Wales (NSW), Australia. The research team also reached out to a Vietnamese women’s health group located in inner city Sydney. Additional participants were recruited through snowballing technique.

To be eligible, participants were required to be over 18 years, identify as Vietnamese, and reside in the state of NSW. Semi-structured interviews were conducted with participants face-to-face, online (via Microsoft Teams/Zoom), or by telephone; depending on participants’ locations and preferences. Interviews lasted about 30–45 min. Participants were provided with AUD $40 cash bank transfer. Interviews were conducted by a Vietnamese-speaking research team member, in English or Vietnamese, depending on participants’ preference. This team member has postgraduate training in health research coupled with rich experience in interview data collection. The interviews, guided by an interview guide (attached as Appendix), explored attitudes and knowledge about health generally and specifically about hepatitis B vaccines, testing, and clinical management; cultural values and beliefs about health, the body, the liver, and hepatitis B; experiences and impacts of stigma; sources of health information; social media use and value given to information on social media; and trusted sources of health information. To ensure cultural competence and appropriateness, the interview guide was reviewed by relevant stakeholders and community organisations. The interviews in English were transcribed verbatim, whereas those in Vietnamese were transcribed and translated by the Vietnamese-speaking researcher. All transcripts and fieldnotes were de-identified and securely stored in UNSW-hosted drive. All participants provided informed consent either verbally or in writing, depending on whether interview was conducted face-to-face or online. The study had ethics approval from the UNSW Human Research Ethics Committee (reference: HC220412).

### Data Analysis

Data analysis was deductive (researcher driven) and inductive (data driven) and conducted in several phases. First, the team developed a data analysis template which aimed to sort, categorise, and summarise the interview data for each participant. Categories included description of health, knowing a person had hepatitis B, knowledge about testing, motivations for testing, knowledge about hepatitis B care and treatment, and health information seeking. Second, a subset of summaries was examined and discussed by the team, and adjustments were made to the categories where necessary. Third, relevant themes were drawn from the codes summarising participants’ insights and perspectives. Five team members coded and analysed the data, and disagreements were resolved through discussion and consultation with senior researchers leading the project.

## Results

### Participant Profile

Participants ranged from 25 to 81 years, and the time in which participants had lived in Australia ranged from 6 months to nearly 40 years. Four participants reported living with hepatitis B, one of whom was on medication. Six participants indicated having an immediate or extended family member living with hepatitis B (see Table 1).

**Table 1:** Demographic characteristics of the sample (*N* = 20)

Socio-demographic characteristics	(*N* = 20)
Gender	
Male	4
Female	16
Age	
18–39	12
40–59	3
60 and above	5
Residence	
Metro	16
Regional/rural	3
Missing data	1
Time living in Australia	
< 5 years	5
5–10 years	6
11–20 years	3
20–25 years	0
25–30 years	1
> 30 years	4
Missing data	1
Hepatitis B status	
Positive and receiving treatment	1
Positive but not receiving treatment	3
Negative and have positive loved ones	6
Negative and does not have positive loved ones	10

### Perspectives and Understandings of Health

Biomedical understandings of health were evident among all participants, whereby they saw the centring of the physical body as the primary site of health and defined health by the absence or presence of physical illness and pain. These participants also perceived indicators of health to be those that are physically observable or measurable, such as blood pressure, body mass index, and biomarkers, and drew on medical concepts and terminology. For these participants, taking a biomedical approach to health would assume that a person’s health is intervenable and can be regulated through behavioural and medical intervention, often at the individual level.

Most participants believed health to be broader than biomedical definitions and talked about more holistic views of wellbeing. These participants spoke to the importance of mental and emotional health, physical exercise, the necessity of social connection and relationships, and how broader social, environmental, and structural factors could impact health. Examples of these factors included air pollution, healthcare systems, cultural health norms, health education. Some participants referred to the popularity and use of exercise and meditation within Vietnamese communities, which they perceived to fit within a holistic health model.

I think a healthy person would spend time to exercise daily, like jogging or walking or swimming… Then mentally, they can also meditate, do yoga, be mindful of their breath. And, they need to nourish their brain by reading or absorbing information from good sources. That’s how holistic health is to me (female/69 years).

Some participants understood health in relation to one’s ability to ‘function’ in their everyday lives, which included notions of productivity, such as through working full-time, as well as being physically mobile, independent, and capable of fulfilling various desires, needs, and expectations. This perspective frames health as necessary for societal stability and is therefore linked to a person’s ability to contribute and to meet certain societal and familial norms and expectations around health, behaviours, duties, and so on.

A healthy person is someone who can do normal activities, eats and sleeps reasonably, has good physical and emotional wellbeing. They can perform some intense activities but can also choose to recuperate whenever they want to reproduce labour power and then can re-engage in work. A person who is not healthy is someone who cannot reproduce labour power physically and mentally, even after resting (female/54 years/living with hepatitis B).

Among some participants was a sense that many people had reactive approaches to health management, which resulted in only seeking medical intervention when they experienced health issues. Participants perceived that many only become health conscious as they get older and begin to experience more health problems.

I think health education in Vietnam is not as well developed. Health-conscious people usually belong to the 30–40 age bracket. Only when they have health-related issues do they start looking after their health. But when they are in their 30s or younger and having no health issues, they would not care much (male/30 years).

Other participants spoke about some people having a more proactive approach to health, with a focus on the prevention of illness and injury. People who took this approach engaged in behaviours to maintain their health and prevent illness, such as eating a healthy diet, engaging in regular exercise, having a good sleep schedule, limiting alcohol and nicotine intake, investing in health education, and going to the doctor for regular check-ups. Perspectives shared by participants suggest that proactive approaches to health were more common among younger people, people living in Australia, and people with higher education due to greater access to resources, health education, healthcare, and community outreach.

### Knowledge of Hepatitis B

Many participants felt that people in the Vietnamese community had limited knowledge about hepatitis B, though they also felt this was true of most communities generally and not just Vietnamese people. As one participant said, ‘I don’t think that we can say [just] Vietnamese in Australia but also, you know, the general public.’ (Participant 4). However, it was generally felt that awareness and education around hepatitis B was improving in the Vietnamese community, due to improved community outreach and access to health information (especially online resources).

The understanding and insight about hep B for Vietnamese people is getting more advanced, it’s getting better - like much more than in the past. Because in the past, … awareness of the people was not really good… But I think now, thanks to the enhanced prevention system, thanks to improved awareness of the people - like people can access social media, leaflets or anything… You know, like on social media, the Internet is much more developed than in the past. So, I think the awareness of the people is getting better and that’s why the testing or like cause or prevention with hepatitis B, they are getting better (male/30 years).

Participants who had hepatitis B or knew someone with hepatitis B tended to be more confident in their hepatitis B knowledge. Overall, these participants had basic knowledge about acute signs and symptoms of hepatitis B, testing, and vaccination. Some participants (often those living with or knowing someone with hepatitis B) were able to refer to acute symptoms and health effects of hepatitis B—including lethargy/fatigue, jaundice, weakened organs/liver, muscle pain, and fatigue. However, some participants were unsure of the symptoms, and only a few participants recognised that hepatitis B could be asymptomatic or ‘invisible’. Most participants were also aware of the availability of a hepatitis B vaccine with many reporting they had been vaccinated—including through work and immigration requirements, routine vaccinations for infants, and being prompted when someone they knew was diagnosed with hepatitis B. A few participants, however, were unsure whether vaccination for hepatitis B existed or if they had been vaccinated.

My female cousin had [hepatitis B]… Back then, she herself told the family to go see GPs for vaccination against hepatitis B. So, I saw the doctor to receive the vaccine – I think 3 doses (female/34 years).

No, I don’t think [there is a vaccination for hepatitis B] because I think it’s like some type of sickness, so I don’t think there will be a vaccine for it (female/30 years).

While some participants were aware of testing via blood test, a few were unsure about how hepatitis B was tested, and some suggested that it could be tested via urine tests or x-rays. A few participants expressed uncertainty about the affordability or accessibility of testing for people in their community—especially for those who were unable to access Medicare (Australia’s universal health care system). This was also noted as a barrier for ongoing management amongst people with chronic hepatitis B.

Even when they’re sick, I don’t think they have any symptoms. So yeah, it really depends. If you want to know if you’ve got the virus, you need to do the blood test. And if you don’t do the blood test, you don’t really know. So, I think that many people may have it and they don’t know that (female/71 years).

Firstly, [a barrier] could be about the cost of testing. Maybe people, they don’t know about how much they have to pay for that and they may be in financial burden. So that’s why they don’t want to take the test (female/36 years).

Participants appeared to have some misconceptions about hepatitis B transmission, prevention (besides vaccination), and treatment/management. Among participants, there was some general understanding that hepatitis B could be transmitted through blood and bodily fluids, including through sexual intercourse, perinatal transmission, and the sharing of unsterilised needles (e.g. tattoo needles, injecting equipment). However, it was also common that people felt uncertainty or held misconceptions about the transmission and cause of hepatitis B, such as believing hepatitis B could be transmitted through sharing eating utensils (e.g. plates, cups, and cutlery) and linen, being near someone infected with hepatitis B, being passed down genetically or resulting from ‘consanguinity’ (relations between two individuals related as second cousins or closer).

It’s mainly due to blood contact too […] And I think that there is kissing then. I don’t think there are other methods of transmission. These days, the government establishes designated injecting centres for the addicts, so that the used needles can be disposed of, instead of being shared around. That’s an approach to prevent spreading of the disease because of sharing syringe (female/81 years).

It might transfer to us during communicating together, like eating together, drinking together with the same cup. Don’t use the same towel and don’t sleep with her. I think that’s the way they transfer (female/30 years).

Participants who did not have hepatitis B had generally limited knowledge about treatment and management. Whilst some participants were aware of the availability of medication to help manage hepatitis B, others were unsure about whether treatment was available. There was also uncertainty about what services were available for people with hepatitis B.

Like myself… I have no idea. Because such information is not easily accessible, especially when we don’t know where or how to find that information (female/34 years).

### Fear and Stigma

Participants reported that hepatitis B was not typically spoken about among the Vietnamese community. Some participants noted negative attitudes toward the virus and stigma as a reason some people might not speak about it. These participants suggested that people living with hepatitis might conceal their diagnosis due to fears of being judged or treated differently, or having their diagnosis impact other aspects of their lives, such as immigration and relationships. Those participants in the sample living with hepatitis B had very real concerns of experiencing stigma and discrimination if they disclosed their hepatitis B status.

In my case, the first time, I felt like not wanting to tell everybody. Maybe because people might judge me. There’d be questions, like, ‘why do you have this virus in your body?’ or maybe ‘you are not a good person, that’s the reason why you have it’. But actually, there are many reasons. So, I think for the information about hepatitis B, it should correct them, explaining [the reasons] very clearly (female/39 years, living with hepatitis B).

I’ve never seen any spontaneous or casual conversations about it… I heard that people having hepatitis B or C may impact [immigration], as they say that someone with hepatitis will cost the government to treat and manage. So, it’s more likely that their visa application will be refused (female/30 years).

Participants thought responses to hepatitis B in their communities varied. However, the stigma that surrounds hepatitis B became apparent, with over half of the participants reporting that they distanced themselves from people living with the virus. This was linked to fears of contagion which were largely based on uncertainty or misconceptions about transmission and seemed to be the biggest driver of hepatitis B-related stigma and discrimination.

To be honest, it did change [relationship with a friend when finding out they were diagnosed with hepatitis B]. We weren’t as close as we had been. We used to play soccer and do most things together, but after that, I kept some distance from him because hepatitis is contagious… In my opinion, if we know a particular person has hepatitis B, we need to keep a distance (male/30 years).

Participants recognised that stigma could negatively impact the lives of people living with hepatitis B. In addition, participants outlined some negative impacts of living with hepatitis B as poorer mental health (e.g. internalising feelings of shame, lowering self-esteem), deterioration of social relationships, and restricted life opportunities due to discrimination, such as barriers to immigration or employment. Participants' perspectives suggest that people with hepatitis B would have significantly more trouble finding romantic partners or a partner for marriage and getting employment as well as restrict them in everyday life activities.

I think they [people with hepatitis B] would have some difficulties. Firstly, they’d have troubles looking for a spouse…. Secondly, studying and doing daily activities are difficult, as others might be able to identify if someone has hepatitis and have a prejudicial mentality and stay away from that person… Even when others are not as prejudiced, the person himself may still have a complex about that, not wanting to interact with people and withdrawing from others (male/30 years).

Some participants mentioned that it was important to reduce the stigma around hepatitis B. These participants further felt that public health education and increasing knowledge particularly about transmission routes were critical to reducing this stigma. In addition, participants mentioned that people living with hepatitis B required additional support to help counter the negative effects of hepatitis B-related stigma and improve their health outcomes.

### Sources of Information

Most participants reported a preference for accessing health information online, such as through social media (e.g. Facebook, TikTok), streaming platforms (e.g. YouTube), government and organisation websites [e.g. NSW health, Ministry of Health, World Health Organisation (WHO), Centres for Disease Control and Prevention (CDC)], news platforms, and other non-official health websites (e.g. WebMD). Government websites, academic sources, and health research and health organisation websites were seen as credible and trustworthy.

Online sources were reported as being more easily accessible, convenient, and an effective way to disseminate health information to a wide audience. The Internet was seen as an easy way to find information in Vietnamese language. However, some concerns were raised about the credibility of information found online and the spread of misinformation. Additionally, some participants (especially older participants) seem to have more difficulty accessing online information due to lower levels of technological literacy and reliability. For these older people, participants perceive that more traditional forms of media—such as newspapers, TV, and radio might be key sources of information, particularly those available in the Vietnamese l.

People like us [women over 50] here would have no clue if the information is online; we would not know how to find it […] I would give up trying to find information virtually, because we don’t know whether it is true or false (female/77 years).

Some participants talked about reading printed health promotion and information materials which was accessed through attendance at health services (e.g. health service/hospital waiting rooms) or through community outreach programs. These types of materials were seen as reliable sources of information and as an effective way to provide health information to people in English and Vietnamese languages. Health practitioners were perceived of as expert sources of health information who were highly credible and trusted and offered personalised advice tailored to the individual. Health practitioners who are Vietnamese-speaking, bilingual, or from CALD backgrounds themselves were viewed as particularly valuable sources of health information for Vietnamese people, because they were seen as understanding the health needs of this community and could communicate in Vietnamese.

I would ask the lady in the women’s health centre - so most of the information that I know, I know from her. Like she will say to me, “have you checked for this test?” [and] because she is speaking to me in Vietnamese, it’s easy for me to understand. And the second place is my GP. He also speaks Vietnamese so he also tells me what to do, to bring the things. Like if my baby is sick, I ask him and he will tell me if we need to go to hospital or not (female/30 years).

My doctor comes from an immigrant background too, so they understand the prevalence of other immigrants who did not have access to vaccination. And they, therefore, are rather knowledgeable… And promotion within the community in Vietnamese, via bilingual Vietnamese doctors and specialists. Because I think people living with hepatitis B would be like me, looking for information in Vietnamese and English (female/54 years living with hepatitis B).

Community outreach was also mentioned by some as an effective and engaging form of health promotion and education—including having stalls to interact directly with people in the community, having health experts, particularly people with lived experience of hepatitis B doing educational workshops and talks with community members and groups, and other outreach interactions by local organisations and community groups.

## Discussion

Understandings of health and wellness among culturally and linguistically diverse communities is key to effectively addressing major public health concerns such as hepatitis B and to develop strategies towards addressing the WHO viral elimination targets set for 2030 [4]. This research contributes to addressing this gap by exploring the health beliefs, healthcare engagement, knowledge and attitudes towards hepatitis B, as well as access to sources of health information among the Vietnamese community in NSW, to inform the development of culturally hepatitis B-related health messages.

Participants drew on multiple understandings of health which have implications for health promotion messaging. Through biomedical and holistic understandings, participants viewed health as something that can, to some extent, be controlled at the individual level such as through adopting healthy lifestyle habits (e.g. diet, exercise, meditation), engaging in health management, accessing healthcare and medical treatment, and accessing heath education resources. Importantly, this suggests that people will respond to calls to intervene in their health if the appropriate and culturally meaningful messages are provided. Health was also measured in terms of one’s ability to ‘function’ in various capacities such as being able to live independently, fulfil desires and needs, be productive citizens, and these views provide some insights into what motivates people to invest in their health. Other research that we have conducted supports the significance of being a ‘productive citizen’ in Vietnamese culture where negative attitudes towards hepatitis B was expressed by the participants as being related to a perceived lack of productivity [26]. Participants also described reactive approaches to health as relatively common in their communities which may be a risk factor for hepatitis B given that it is often asymptomatic (i.e. ‘invisible’) and thus could go undetected in people who only seek health support if experiencing symptoms of an illness. Hepatitis B is often referred to as the ‘silent disease’ because symptoms do not usually appear until the end stages of liver disease, contributing to low hepatitis B screening rates [27]. This emphasises the need to promote early testing and detection to ensure that people have regular routine monitoring as compared to symptom-driven interventions. Efforts to improve community outreach and accessibility to health education and health care should involve promoting more proactive approaches to health care and management, which would then encourage improved hepatitis B prevention strategies, including earlier detection and monitoring [28–30].

Findings from the study also support previous research that shows that knowing someone with hepatitis B is linked with better knowledge around transmission routes, prevention and treatment [26]. Given the relatively high prevalence of hepatitis B among the Vietnamese community, it is important to improve knowledge about hepatitis B. Literature on hepatitis B among CALD communities continues to show that hepatitis B knowledge is low or inconsistent and that there is confusion about vaccinations, transmission routes, and between the different types of viral hepatitis [25, 31, 32]. Though low knowledge has been identified as a key driver of negative attitudes towards hepatitis B, with increased knowledge associated with more positive attitudes [8, 9, 13]; negative attitudes may still co-exist with high knowledge [33]. Our study shows that stigmatising attitude towards hepatitis B exist in the community and negatively impacts people living with hepatitis B. This finding is supported by previous research within Vietnamese communities, where stigma regarding hepatitis B, including guilt and shame, were found to be a barrier to prevention, testing, and treatment [13, 34]. This highlights the importance of providing targeted health promotion initiatives and resources to the Vietnamese communities in Australia to increase understanding of transmission, reduce stigma, and improve access to healthcare, especially since previous research has found that increased knowledge is associated with more positive attitudes and better trust in Western healthcare [26]. Additionally, since knowing someone living with hepatitis B has been found to increase knowledge and reduce stigma [9, 26], hepatitis B intervention programs with people with lived experience from the Vietnamese community can further be utilised to increase knowledge and reduce stigma and negative attitudes.

Implications from this study for future hepatitis B prevention and care efforts include developing culturally and linguistically appropriate educational resources and programs to increase knowledge and decrease stigma in the community [12], providing resources to support healthcare workers in delivering hepatitis B testing and care to Vietnamese patients in culturally appropriate ways [23, 35], and exploring the potential of web-based education campaigns—especially for reaching younger people [32]. In terms of mediums for health promotion, online resources seem to be preferred by more participants; however, participants did recognise that older people would use these less. Online resources were valued because they were more easily accessible and convenient, and information in the Vietnamese language could be easily found. Participants reported using a wide range of online sources but preferred official and expert sites where credibility and legitimacy could be trusted, such as government websites (state and national), academic sources, and health organisation websites (e.g. WHO, CDC). Printed materials in health care or through community outreach were generally seen to be trustworthy, although not as widely and conveniently available, and traditional media in the Vietnamese language was also viewed as a valuable source of health information for older people.

Participants felt that advice and information received by talking with other people was viewed as useful; however, this was only perceived of as valued if the advice came from a source that was deemed credible such as doctors or medical professionals, or people with lived experience. Medical professionals of the same background as participants were particularly valued, and aside from being able to communicate in Vietnamese if preferred, participants also felt that these health workers would be able to identify with them and understand the experiences and difficulties that people of CALD backgrounds may face. Research among Chinese people living with hepatitis B in Australia highlights the importance of connection and trust between health workers and clients [9]. Studies have shown that disparities in health beliefs systems between a clinician and patients as well as a dismissive approach to traditional health beliefs undermines trust in healthcare providers and hence supports the notion that health workers of the same background as patients, or at minimal who understand and accept the culture and migration experiences of CALD communities, is very important in building rapport and ensuring health messaging is accepted by CALD communities [9, 35, 36].

This study’s results should be understood within some caveats. The sample is self-selected, and recruitment was challenging, even a sample of twenty participants was difficult to obtain and required various recruitment strategies. It is likely that those who may have more difficulty talking about hepatitis B or less knowledge about hepatitis B may not have volunteered for an interview. On the other hand, a strength of this study lies in the fact that one of the researchers is of Vietnamese background, thus ensuring that interviews could be conducted in Vietnamese if requested. In addition, this researcher of Vietnamese background collaborated in the analysis and understanding of the data ensuring that cultural understandings were appropriately attended to. Additionally, the sample includes four people living with hepatitis B who provided valuable information about their experiences. The findings of this study provide important insights into the perceptions of people from Vietnamese backgrounds in Australia around hepatitis B, including understandings of health, knowledge of the virus, attitudes towards hepatitis B and which sources of health information are trusted by the community. By understanding what Vietnamese people know about hepatitis B as well as how they access their health information, the findings of research can be used to inform the design of health promotion campaigns using multiple mechanisms to ensure wide reach of these messages, such as via social media, print media, and radio or television programs. In addition, education resources must be accessible in a range of languages and delivered in places or by people perceived of as credible and trustworthy to community. Knowledge of this community specific information is key to reducing the burden of hepatitis B among CALD communities and ensuring they are comfortable to access healthcare services for hepatitis B testing, monitoring, and care.

## Appendix Interview Guide

### Background

*Can you please tell me a little about yourself?*

- How old are you?
- Where do you currently live?
- What gender do you identify with?
- Where were you born?
- Where did you grow up?
- For those born in Australia:
  - How long has your family been in Australia?
  - How did your family come to be in Australia?
- For those born outside Australia:
  - How long have you been living in Australia?
  - How did you come to be in Australia?

### Values about Health and the body…

*Before we discuss hepatitis B, I’d like to briefly ask you the idea of ‘health’ more generally. We are interested in knowing how people understand health and being healthy*:

- What does a healthy person look like to you?
- Among the Vietnamese people you know in Australia, how do people look after their health?
- Do you think health is understood differently in Australia compared to Vietnam?

### About Living with Hepatitis B…

We are interested to hear about any personal experiences with hepatitis B.

- do you or anyone close to you have hepatitis B?

If you have hepatitis B:

- In what circumstances, and where, did you first find out that you had hepatitis B?
- Can you describe how you found out that you have hepatitis B?
  - Who told you? Why did you get tested? Where did you get tested? If at a medical service what were the staff like?
- In your opinion what would encourage people to come forward to get tested for hepatitis B?
- Who knows that you have hepatitis B — family, friends, colleagues? Did your relationship change because of your disclosure?
- Can you tell me what kind of treatment you have received for your hepatitis B?
  - What sort of health care services have you been to about hepatitis B?
  - How long you’ve been getting treatment?
  - How did you find out about this service in the first place?
- With the services you have seen about hepatitis B, how helpful are they for Vietnamese people?

If someone close to you has hepatitis B:

- How do you know that they have hepatitis B?
- Do you talk about hepatitis B and medical treatments with them? What do you talk about?
- Do you help them with their treatments? In what ways do you help them?

### Knowledge about Hepatitis B Prevention and Health care…

*About testing*:

- How do people know that they have hepatitis B?
- In your opinion, do the Vietnamese people you know understand what is involved in getting tested for hepatitis B?
- Are people worried about the testing process and why?
- What might keep people from getting tested?

*About health care for hepatitis B*.

- Where do the Vietnamese people that you know go to seek help about hepatitis B?
- What would stop people from seeking help about hepatitis B?
- Do the Vietnamese people you know have an understanding of what’s involved in the treatment for hepatitis B?
- What do Australian health services need to understand when trying to connect with Vietnamese Australians about hepatitis B?

*Stigma*:

- In your opinion is hepatitis B something that the Vietnamese people living in Australia talk about or are concerned about? Why or why not?
- If someone has hepatitis B, what sort of difficulties does this bring to their lives? For example with work, education, marriage, family relationships.
- What are people’s attitudes towards someone with hepatitis B in the Vietnamese community?

### About Health information…

*We want to know more about how people in the Vietnamese community get information about health and more specifically about hepatitis B*:

- What are the most common ways that you learn about your health or find out health-related information? (for example think about how you found out about COVID vaccines or flu vaccines)
  - From friends, family or other people?
  - TV or online sources?
  - Facebook or other social media?
- What sources of health information do you trust most?
- What sources of health information are most trusted by the Vietnamese people you know in Australia?
- Is this different for the older and younger generation? Or for men and women?
- Where do you (or where would you) get information about hepatitis B? Where do other Vietnamese people in Australia get information about hepatitis B?
  - From friends, family or other people?
  - TV or online sources?
  - Facebook or other social media?
- What sources of information about hepatitis B would/do you trust most?
- What sources of information about hepatitis B would be most trusted by the Vietnamese people you know?
- Is this different for the older and younger generation? Or for men and women?
- In your opinion, what is the best way for Vietnamese people in NSW to receive health promotion messages about hepatitis B? Social media, newspapers, other methods?
- What kind of information about hep B do you think needs to be on social media?

Close: Is there anything that you’d like to say that I haven’t asked you about?

Is there anyone you know who might be willing to be interviewed, and would you be willing to pass on the study information to them?
